# Cancer-Associated Fibroblasts: The Origin, Biological Characteristics and Role in Cancer—A Glance on Colorectal Cancer

**DOI:** 10.3390/cancers14184394

**Published:** 2022-09-09

**Authors:** Charalampos Fotsitzoudis, Asimina Koulouridi, Ippokratis Messaritakis, Theocharis Konstantinidis, Nikolaos Gouvas, John Tsiaoussis, John Souglakos

**Affiliations:** 1 Laboratory of Translational Oncology, School of Medicine, University of Crete, 70013 Heraklion, Greece; 2 Department of Nursing, Hellenic Mediterranean University, 71410 Heraklion, Greece; 3 Medical School, University of Cyprus, 20537 Nicosia, Cyprus; 4 Department of Anatomy, School of Medicine, University of Crete, 70013 Heraklion, Greece; 5 Department of Medical Oncology, University Hospital of Heraklion, 71110 Heraklion, Greece

**Keywords:** cancer-associated fibroblasts, CAFs, tumor microenvironment, colorectal cancer

## Abstract

**Simple Summary:**

Tumor microenvironment is a major contributor to tumor growth, metastasis and resistance to therapy. It consists of many cancer-associated fibroblasts (CAFs), which derive from different types of cells. CAFs detected in different tumor types are linked to poor prognosis, as in the case of colorectal cancer. Although their functions differ according to their subtype, their detection is not easy, and there are no established markers for such detection. They are possible targets for therapeutic treatment. Many trials are ongoing for their use as a prognostic factor and as a treatment target. More research remains to be carried out to establish their role in prognosis and treatment.

**Abstract:**

The therapeutic approaches to cancer remain a considerable target for all scientists around the world. Although new cancer treatments are an everyday phenomenon, cancer still remains one of the leading mortality causes. Colorectal cancer (CRC) remains in this category, although patients with CRC may have better survival compared with other malignancies. Not only the tumor but also its environment, what we call the tumor microenvironment (TME), seem to contribute to cancer progression and resistance to therapy. TME consists of different molecules and cells. Cancer-associated fibroblasts are a major component. They arise from normal fibroblasts and other normal cells through various pathways. Their role seems to contribute to cancer promotion, participating in tumorigenesis, proliferation, growth, invasion, metastasis and resistance to treatment. Different markers, such as a-SMA, FAP, PDGFR-β, periostin, have been used for the detection of cancer-associated fibroblasts (CAFs). Their detection is important for two main reasons; research has shown that their existence is correlated with prognosis, and they are already under evaluation as a possible target for treatment. However, extensive research is warranted.

## 1. Introduction

Solid tumors and their treatment are an ongoing challenge for scientists. In recent years, tumor microenvironment (TME) seems to have an emerging role in tumorigenesis, tumor growth, metastasis and resistance to therapy [1,2]. TME consists of the extracellular matrix (ECM) and different cells and micromolecules (tumor cells, immune cells, vasculature and cancer-associated fibroblasts (CAFs)) [2,3]. All of them are involved in tumor growth, mainly by activating different pathways, and provide information on progression to one another [4,5]. Fibroblasts are the main stroma components leading to ECM remodeling in the connective tissue [6,7]. They are mesenchymal cells that can be differentiated under specific signals in various organs [3]. CAFs are fibroblasts with a crucial role in tumor growth and metastasis by interacting with cancer cells via various mechanisms, such as exosomes, production of cytokines or cell-to-cell contact [8]. Although their detection and characterization are not apparent, they seem to be prognostic biomarkers of immense importance with therapeutic implications [8,9].

Despite the progress achieved in cancer therapeutics, colorectal cancer (CRC) remains an important health issue. It is the third most frequent cancer worldwide, and its incidence is increasing, especially in the Western world [10]. The treatment options include chemotherapy and, uncommonly, immunotherapy, as a choice in a specific number of patients (maximum 15% in stage IV) [11]. In the aspect of translational oncology, four molecular subtypes have been announced by the CRC Subtyping Consortium (CRCSC): CMS1, CMS2, CMS3 and CMS4 [12]. The criteria for this categorization were: transcriptomic profiling, microsatellite instability, mutation characteristics, somatic copy alternations number and DNA methylation. The CMS4 subtype (~20%), known as mesenchymal, is the most relevant to TME and ECM reconstruction, with the worst prognosis [12]. However, until now, an important part of TME has been omitted. CAFs seem to have a prognostic significance at every stage of carcinogenesis and tumor growth in CRC [4,8,9]. Wu J et al. [13], in a systemic review and meta-analysis interpretation, have shown that the existence of CAFs in the stroma around colorectal adenomatous polyps and the primary tumor site is related to poor prognosis and higher recurrence rates.

## 2. Pro-Tumor Effects of CRC-Associated Fibroblasts

Tumor-cell-derived Nodal stimulates the transition of normal fibroblasts into CAFs, which function to enhance the tumor growth of CRC cells, both in vitro and in vivo, by activating the TGF-β/Smad/Snail pathway [14]. Snail-positive fibroblasts present CAF properties [15], supporting the hypothesis that Snail is a critical controller of CAF arrangement determined from the fibroblasts. Snail could be a TGF-β target gene that intercedes with a few pro-tumorigenic roles in TGF-β signaling [16,17] and is additionally essential for inducing the pro-tumorigenic impacts of fibroblasts on CRC cells [18]. It is subsequently sensible to conjecture that Nodal-mediated CAF arrangement through Snail signaling seems to advance forceful phenotypes in CRC. Moreover, apart from the Nodal, interleukin (IL)-34, a cytokine overexpressed by CRC cells, can also fortify ordinary fibroblasts to present a cellular phenotype [19]. Hence, the crosstalk between CRC and fibroblasts induced by solvent variables, such as Nodal and IL-34, plays a critical part in upgrading CAF arrangement within the TME of CRC. Moreover, other CRC cell-secreted components may likely take an interest in directing the separation of fibroblasts into CAFs, which warrants further investigation.

A few up-to-date studies have also demonstrated the essential functions of cancer stroma in the improvement of CAFs in CRC. For instance, the expanded stromal expression of the tissue inhibitor framework metalloproteinase-1 (TIMP-1) fortifies the collection of CAFs inside CRC tissues somewhat through trans-differentiation of the inhabitant fibroblasts [20]. Furthermore, dickkopf-3 communicated within the stroma organizes a concomitant actuation of Wnt and YAP/TAZ signaling, which are facilitated to produce CAFs in CRC [21]. Additionally, the stromal loss of protein kinase Cζ (PKCζ) advances an era of a pro-tumorigenic CAF populace in human CRC through a SOX2-dependent mechanism [22].

### 2.1. Origin of CAFs

**Figure 1:**
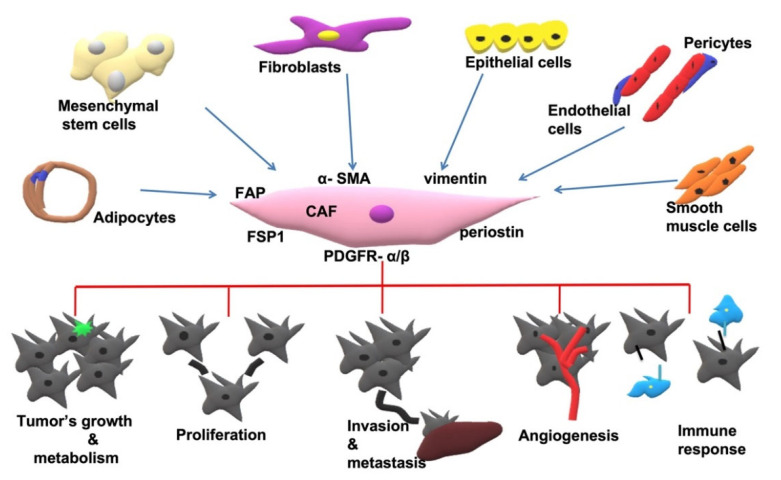
Origin of CAFs and their relation to cancer. CAFs might be derived from mesenchymal cells, fibroblasts, epithelial cells, endothelial cells, adipocytes, pericytes and smooth muscle cells. The main markers that can be recognized regarding GI cancers are: FAP, α-SMA, vimentin, FSP1, PDFGR-α/β and periostin. They are related to cancer by promoting tumor growth, cancer cell proliferation, invasion and metastasis, angiogenesis and immune remodeling.

Concerning their origin, CAFs could be derived from: (i) mesenchymal stem cells (MSCs) [23,24,25,26]; (ii) migration of circulating fibrocytes into the TME [27,28]; (iii) epithelial-to-mesenchymal transition (EMT) [29]; (iv) endothelial-to-mesenchymal transition (EndMT) of resident endothelial cells [30]; and (v) (less common) trans-differentiation of adipocytes, pericytes and smooth muscle cells (SMCs) (Figure 1) [31].

In CRC, fibroblasts are considered the main sources of CAFs, although their origin is not well known [32,33]. The differentiation of quiescent fibroblasts in the TME rapidly becomes activated when stimulated by a convention protein, TGF-β. The trans-differentiation of those resident fibroblasts into CAFs is induced by the increased TGF-β production within the TME, which is triggered by the induction of CRC cell-derived soluble factors [34]. Among the ways TGF-β is activated, it has been revealed that the integrin alpha-v beta-6 (ανβ6), which is expressed in CRC cells, is responsible for the activation of the latent form of TGF-β. This is crucial for the exhibition of the CAF phenotype, as in the absence of this, the activation of the latent form of TGF-β is ruptured [35]. All of this leads to the importance of the capability of activation of TGF-β and the activation of CAFs in CRC.

Furthermore, Nodal, which is a TGF-β superfamily member, critical for endomesodermal induction, is associated with α-smooth muscle actin (α-SMA) positive expression in CRC tissues and contributes to the transition of resident fibroblasts into CAFs through the activation of TGF-β/Smad/Snail pathway [14]. In addition to Nodal, it was found that cytokines (IL-34) are expressed in high abundance in CRC cells, which are also involved in the transition of normal fibroblasts into CAFs [19]. Based on various studies, tumor stroma plays an important role in the evolvement of CAFs in CRC. Gong et al. [20] demonstrated that the augmentation of stomal expression of the tissue inhibitor matrix metalloproteinase-1 (TIMP-1) leads to the stimulation of accumulation of CAFs, which are trans-differentiated by normal fibroblasts. Moreover, Ferrari et al. [21] revealed that the activation of Wnt signaling and YAP/TAZ signaling by the increased stromal expression of the secreted glycoprotein dickkipf-3 also leads to the increase in CAFs. Additionally, Kasashima et al. [22] found that the promoted tumorigenic function of CAFs is strongly associated with stromal loss of protein kinase Cζ (PKCζ). Among other sources responsible for the origin of CAFs, Peng et al. [36] described the differentiation of mesothelial cells into CAFs by the cell-to-cell interaction, which is mediated by Notch-Jagged1 signaling and downstream activation of TGF-β/Smad pathway. Wawro et al. [37] revealed that TGF-β-dependent phosphorylation of tubulin-β3 gives rise to CAFs via endothelial-to-mesenchymal transition (EndMT) of endothelial cells. It is not clear how mesenchymal stromal cells differentiate into CAFs via mesothelial-to-mesenchymal transition (MMT). Based on RNA sequencing, Rynne-Vidal et al. [38] demonstrated a correlation of TGF-β signaling and MMT. Despite the above-described precursor cells that are sources of CAFs in CRC, it is well accepted that the mesenchymal stromal cells can also differentiate into other mesenchymal-like osteocytes, chondrocytes and adipocytes. This is why upcoming techniques have to be employed to identify precisely the origin of CAFs in CRC as a strong tool of increased knowledge of the complexity of CAFs in CRC.

### 2.2. Tumor Microenvironment and Fibroblast Heterogeneity

It is well known that cancer is not just an aggregation of abnormal cells growing uncontrollably. Cancer constitutes an organ encompassing a wide range of several cell populations, which coactivate between the cell population, the tumor cells and their own microenvironment. TME plays a crucial role in the initiation, proliferation and metastasis of a tumor. Its major components are the cells themselves and the ECM, which supplies a structural component of the TME. CAFs are the activated fibroblasts of the stroma and secrete growth factors, inflammatory ligands and ECM proteins [39,40,41,42,43,44].

The capacity of CAFs to create significant amounts of ECM proteins, including collagen, glycoproteins and proteoglycans, is one of their key characteristics [45]. Under healthy and pathological circumstances, the ECM serves as a complex structure that anchors and supports environmental cells via hundreds of different proteins [46]. Collagens, proteoglycans and hyaluronic acid are important ECM structural components that provide a framework within cells, and other ECM elements (such as laminin or fibronectin, for example) interact with them [45,47]. Moreover, ECM proteins transmit signals to cells, which are then processed by integrins and other cell surface receptors. The cellular pathways activated by these signals, known as mechano-transduction, have an impact on cellular processes, such as proliferation, survival, morphology, adhesion and motility [48,49,50]. Due to the matrix protein ability to sequester growth factors and change their signaling properties, the ECM also serves as a growth factor reservoir [51]. The biochemical composition, mechanical characteristics and integrity of the ECM are frequently altered in conditions such as fibrosis, cardiovascular or musculoskeletal diseases [52,53,54] and cancer [55]. Recently, it was demonstrated that ECM possesses biomechanical and physical characteristics that affect all cancer hallmarks, including cellular processes that contribute to tumorigenesis, development and spread [56] and metastatic potential [57,58]. Additionally, the pancreatic ductal adenocarcinoma constitutes up to 90% of the tumor region [59], and the fibrotic stroma in breast cancer is unusually dense, which hinders blood flow to the tumor and the delivery of anticancer medications [60]. More aggressive tumors and a worse prognosis for the patient have been demonstrated to be correlated with ECM alterations within tumors (quantity, stiffness, etc.) [61,62,63,64].

Το define CAFs, we need to understand normal fibroblasts, whose definition remains unclear due to the scarcity of specific markers that are not expressed in any other cell types, such as adipocytes, chondrocytes and osteoblasts. All of them share the same embryonic origin (from the primitive mesenchyme that develops out of the mesoderm) [65,66,67,68]. In practical terms, to define fibroblasts, their cell shape, location and the lack of markers for epithelial cells, endothelial cells and leukocytes are evaluated.

In non-cancerous homeostatic conditions, fibroblasts are the main producers of the connective tissue ECM, presenting at the resident stage and exhibiting an important sensor of tissue integrity [69]. The resulting data indicate that this function has a strong relation with age [70,71]. Following the tissue damage signal, the activated fibroblasts—termed as myofibroblasts—regulate tissue repair, participating in a crosstalk with immune cells [72,73]. Their role in wound healing is to contract the wounds while producing and organizing the ECM [74]. Throughout the time when the wound heals and the scar is formed, the myofibroblasts are eventually disappeared after they became apoptotic [75].

The spectrum of differences between normal fibroblasts and myofibroblasts comprises (a) an active endoplasmic reticulum [76], (b) expression of α-SMA and increased levels of vimentin [77], (c) the arrangement of complex and organized stress fibers and fibronexus adhesion complexes [78]. There is formulation of a permissive action in the interaction between the bundles of microfilaments and the ECM proteins that preserve the cellular contractile force and allow the fibroblasts to maintain their microenvironment. This results in the production of ECM proteins (such as collagen, elastin, fibronectin, tenascin and remodeling enzymes) [79,80].

In cancerous conditions, the cancer cells contort the process of wound healing, and they have the ability to migrate away from or infiltrate into adjacent tissues. CAFs constitute the activated fibroblasts of the stroma and secrete growth factors, inflammatory ligands and ECM proteins. The difference between normal fibroblasts and CAFs is the expanding production of collagen and ECM protein, which activates them in a hyper state that magnifies cancer progression.

To support a tumorigenic primary niche, many stromal cell types congregate. Tumor cells are exposed to immune-system-driven demands for elimination after evading the cell-intrinsic processes of apoptosis [81]. This process involves tumor-cell-specific antigens, which are identified by cytotoxic immune cells and result in the elimination of tumor cells [82]. Within the TME, fibroblasts and macrophages also help restrict growth, but the tumor may educate these cells to develop pro-tumorigenic properties [83]. For example, by secreting an abundance of pro-tumorigenic proteases, cytokines and growth factors, TAMs support a variety of behaviors within the primary tumor, such as growth, angiogenesis and invasion (e.g., EGF, which participates in a paracrine signaling loop via tumor-secreted CSF-1) [84]. Immune-suppressive cells, such as myeloid-derived suppressor cells (MDSCs) and regulatory T (Treg) cells, are released into circulation as the tumors spread in response to activated cytokine axes brought on by carcinogenesis (e.g., TGF-, CXCL5-CXCR2) [85,86,87,88,89]. Invading the expanding tumor, MDSCs and Treg cells interfere with immune surveillance via a variety of means, such as by preventing DCs from presenting antigen, by preventing T- and B-cell proliferation and activation or by preventing NK cytotoxicity [90,91,92]. CAFs, which are activated by factors from the tumor (such as TGF-, FGF, PDGF, etc.) [93,94], release ECM proteins and parts of the basement mem-brane, control differentiation, modify immunological responses and contribute to dysregulated homeostasis [95,96]. Vascular endothelial growth factor (VEGF) promotes angiogenesis during tumor growth and is mostly derived from CAFs [97]. In addition to cellular influences, several extracellular factors, such as low oxygen tension, high interstitial fluid pressure and modifications in particular ECM components, aid in the tumor progression [98,99].

### 2.3. Markers Expressed in CAFs

There are few markers that need to considered within the tissue position and their morphology to distinguish CAFs from normal fibroblasts, such as vimentin and platelet-derived growth factor receptor-α (PDGFRα). For some fibroblast subtypes, such as those that characterize bone and fat homeostasis, the markers include α-SMA and fibroblast activation protein (FAP) [100]. According to various studies, there are several limitations in the specificity and usability of CAF markers. Not only is there a lack of adequate exclusion criteria between other mesenchymal lineages (such as pericytes or adipocytes) and an unclear correlation with specific cancer types/positions, but the experimental studies are also limited. Hence, the upcoming studies are not only focused on identifying new markers but also novel methods for selecting CAFs, while analyzing them based on cellular function [100,101,102,103].

Since CAFs are found in a wide range of tumors (Table 1), each CAF plays a specific role. It is well established that CAFs amplify their heterogeneity. Thus, the markers are not all expressed similarly or concurrently in CAFs.

The lack of specific markers for CAFs in numerous cancers is also the case for CRC. However, the effort made over the recent decades has led to a progress in the development of a few markers of CAFs in CRC and clarifying their relationship with tumor growth, proliferation, migration and therapy resistance. Among others found in numerous cancers, cell-surface molecules CD10 and IL-11 could be considered as markers of CAFs in CRC. Since the expression of these molecules is not at the same levels in CAFs and normal fibroblasts, they might be used as biomarkers [104,105]. An entire set of proteins constitutes the profile of CAFs as biomarkers, including LTBP2, CDH11, OLFML3 and FSTL1 [106]. Additionally, based on histological examination from samples of CRC patients, an increased expression of disintegrin and metalloproteinases (ADAMs) has been detected [107]. Furthermore, according to Herrera et al. [108], a major number of ncRNAs using NGS could also be identified as biomarkers. Studying a set of proteins expressed in the specimens of CRC patients and secreted into the extracellular space of CAFs and bone-marrow-derived precursors, De-Boeck et al. [109] demonstrated a few possible biomarkers, such as tenascin C, fibronecrin ED-A domain and stromal-derived factor-1 (SFD-1).

Collagen I, PDGFR-β and α-SMA play a significant well-known role in CRC progression, and in advanced stages, they are correlated with vessel markers CD31 and CD34 [110]. Furthermore, Sugai et al. [111] revealed the relationship between a few CAFs markers (α-SMA, CD10, podoplanin and FSP1) and lymphatic metastasis in submucosal invasive CRC. As these markers were expressed predominantly in stroma-high CRC samples, they could have a valuable predictive and prognostic impact [112].

### 2.4. CAFs Implementation in Cancer

#### 2.4.1. CAFs Promote Tumor Growth

As mentioned above, CAFs are a major component of stoma, and they participate in cancerous conditions by the secretion of cytokines, growth factors and chemokines while becoming activated by tumor and/or non-cancer cells (paracrine signaling). According to studies, the interaction of tumor cells and CAFs represents an important role in the tumor progression. This interaction activates CAFs via a number of factors and their own receptors, such as the hepatocyte growth factor (HGF), transforming growth factor-beta (TGF-β), stomal-derived factor 1 (SDF-1), interleukin 1β (IL-1β), PDGF, phosphatase and tensin homolog (Pten) and Sonin Hedgehog (Shh) [113,114]. For that reason, CAFs are considered as a tumor-promoting component, supporting the growth, proliferation and migration while developing therapy resistance and excluding immunity. Additionally, through the secretion of growth factors, an enhancement of stem-cell-like properties is observed, which plays an important role in tumor formation. The conservation of these properties participates in the regulation of differentiation and proliferation of cancer stem cells by providing a supportive TME [115]. The tumor promotion function of CAFs has been studied in numerous cancers; however, our knowledge is still limited. There are various regulators and pathways that have been revealed in mice models. The AOM/DSS mouse model, which is an initiation promotion model, was used by a few researchers in an effort to reveal the role of CAFs in oncogenesis [116,117,118,119]. Yuan et al. [120] described the participation of MyD88 signaling in myofibroblasts in the activation of the STAT3/PPARγ pathway and the resistance of the myofibroblasts. The MyD88-deficient mouse model resulted in the induction of tumorigenesis. The activated STAT3 exhorted specific ECM proteins to accelerate CRC development in the AOM/DSS mouse model. This played a crucial role in tumor growth because it was found that its promotion was embedded with the activation of STAT3 in CAFs in CRC [121]. Moreover, its inactivation restricted such tumor promotion [122]. Additionally, the production of CAFs by activated transcriptional factors (SOX2), as it was associated with the loss of PKCζ promoting intestinal tumor growth, as described by Kasashima et al. [22], was involved in tumorigenesis. Furthermore, specific genes were significantly correlated with CRC tumor development, as they regulated specific signaling pathways [123].

#### 2.4.2. CAFs and Angiogenesis

Another mechanism through which CAFs participate in tumor malignancy is by conducting angiogenesis. They improve nutrient, oxygen and growth factor supplementation of tumors through growing blood vessels. The expression of the connective tissue growth factor (CTGF) and SDf-1/CXCL12 results in directly increased microvessel density and recruitment of endothelial cells, respectively [124]. On the other hand, the expression of MMPs (such as MMP9 and MMP3) results in the release of active growth factors, such as VEGF, which indirectly increase the tumor angiogenesis [125,126].

Based on studies, the function of CAFs is controlled by the CAF-secreted chemokines, and their chemokine co-receptors play an important role in tumor growth in numerous cancers (breast, skin, head and neck, RCC, GI). In breast cancer, the CAF-secreted SDF-1 comes along with the loss of mDia2 protein expression, contributing to the acceleration of tumor cell growth. This was found to modulate a function of CAFs, through which they recruit endothelial progenitor cells into breast cancers. This results in the promotion of vascularization [127,128]. Additionally, the CAF-secreted VEGF participates in the formation of vascularization to further promote tumor growth and outspread [129].

The limited data regarding head and neck cancer demonstrate that the CAFs are associated with the formation of new blood vessels in nasopharyngeal carcinoma, as elevated CAFs marker α-SMA levels were found in the stroma. The measurement of tumor angiogenesis was higher in the stroma of the cancer tissue (as the positivity of CXCR4 (receptor of SDF-1) and CD133/VEGFR-2 cells points out). The existence of endothelial progenitor cells in both the tumor tissue and stomal cells means that there is a form of VEGF- and SDF-1-dependent vascularization [130].

Furthermore, limited to skin cancer, Erez et al. [131] demonstrated that CAFs are implemented both directly and indirectly in neoangiogenesis—directly through the expression of genes (such as CYRR2 and OPN) and indirectly through the expression of inflammatory genes (such as CXCL10, CXCL2 and CXCL5). The accumulation of HIF-1a promotes the activation of CAFs in hypoxic conditions via SDF-1 secretion by α-SMA-positive CAFs, leading to angiogenesis in RCC [132,133]. In gastrointestinal cancers, and particularly in hepatocellular carcinoma (HCC), increased levels of α-ASMA and THY1 expression in stroma and peritumoral tissue are associated with enhanced levels of placenta growth factor (PGF) expression, which, together with CD90, provide a strong correlation with tumor angiogenesis markers (CD31, CD34, CD105) [134].

Comparing the secretome of CAFs and normal fibroblasts, it was demonstrated that the strong involvement of CAFs in the proliferation of CRC cells promoted tumor growth both in vitro and in vivo [14,135]. The secreted periostin, a multifunctional ECM protein, the active participation of CAFs in the metabolism of tumor cells and the mediated IL-6 could elucidate the malignant proliferation of CAFs [136,137,138]. Further indications of the promoting proliferation functions of CAFs on CRC acting as mediators include the microRNA-31, ncRNA UCA1 and a few signaling pathways, such as PI3K-Akt, FGF-1/-3/FGFR4 and ERK5/PD-L1 [139,140,141,142,143]. As previously mentioned, tumor needs nutrients and oxygen to survive. It is well accepted that the vascularization of cancer plays a pivotal role in this but also in its growth, proliferation and migration. In CRC, IL-6 participates in angiogenesis via increased VEFG production, which is an important source of IL-6 [144]. Heichler et al. [145] revealed that the transcription patters regulated by IL-6-activated STAT3 are of high importance in tumor vascularization and, by inhibiting this pathway, could disrupt CRC growth [122]. Moreover, the Wnt signaling pathway promotes enhanced tumor angiogenesis in CRC.

#### 2.4.3. Invasion and Metastasis

In addition to the local mechanisms by which CAFs participate in tumor growth, supplementation and vascularization, CAFs are responsible for facilitating metastases to distant organs [114]. Both head-to-head cell interactions and secreted factors (cytokines, chemokines, inflammatory mediators) can incite cancer invasiveness [115]. Cancer cells lying in the stroma can migrate to other sites through paths and tracks that are created by CAFs via proteolytic and structural amendment, mediated by proteins and enzymes [146,147]. Further detailing cancer invasiveness, the environmental factors (such as proteins) are a component of stromal support for cancer cells that have spread throughout the site of the primary tumor. The induction of such factors can accelerate metastatic colonization of cancerous cells via cytokines signaling, which further enhance the metastatic pattern through the induction of epithelial–mesenchymal transition via paracrine signaling. This results in CAFs acquiring mesenchymal properties to invade and spread elsewhere [148,149].

This is also the case in CRC patients [150]. The secretome of CAFs from distant metastatic sites resulted in more adjectival characteristics of the tumor, such as epithelial-to-mesenchymal transition, invasion and metastasis [151]. The variety of factors secreted by CAFs disrupt the signal transduction pathways between the CAFs and CRC cells, as was discovered in PDGF receptor signaling. Such signaling is mediated by a glycoprotein secreted by CAFs (stanniocalcin-1), resulting in increased invasion and migration of CRC [152]. The secretion of the hepatocyte growth factor (HGF) mediated by Ras-related protein Rab-31 could provide the migration of CRC [153]. The HGF participates in the EMT via the CAFs’ secretome [154]. Additionally, the fibroblast growth factor-1 (FGF1) and stomal-cell-derived factor-1 (SDF-1) could enhance CRC metastasis via FGFR3 signaling and CXCR4 axis, respectively [35,155]. Among the other factors secreted by CAFs and found to play a crucial role in tumor migration, invasion and EMT, CLEC3B, activin A and Wtn2 have been recently recognized [156,157,158]. Interestingly, adjacent cancer cells could be affected by exosomes, such as miR-15-5p and LINC00659, promoting metastasis, invasion and migration of CRC cells [159,160].

Cancer cell metabolism constitutes another aspect of the mechanisms that participate in CRC progression. It is known that the activated CAFs use glutamine as an energy source in CRC cells, which results in multiple organ metastases [161]. Additionally, CAFs activate fatty acid oxidation and regulate glycolysis, both playing a role in peritoneal metastasis and promoting migration and invasion of CRC cells [162].

#### 2.4.4. CAFs and Cancer Cell Metabolism

The Warburg effect, as described by Warburg et al. [163,164], demonstrates an increase in glucose consumption by the tumor cells and a preferential production of lactate, even in the presence of oxygen. Based on this, numerous studies described that cancer cells are able to synthesize lipids, amino acids and nucleotides while facilitating tumor cell growth, proliferation and migration [163,164]. The discovery of signal transduction pathways between the CAFs and cancer cells, based on an increased knowledge of cancer metabolomics, revealed different metabolic pathways across cancers, implying ECM stiffening and autophagy. The suggestion that CAFs also exhibit the Warburg effect comes from a number of studies in CAFs isolated from different cancer types (such as breast, colon cancer, pancreatic cancer, melanoma, lung cancer). The cancer cells provide aerobic glycolysis in CAFs, which produce increased levels of pyruvate and lactate to be used as an energy source [165,166,167,168]. Isolated CAFs from various cancers demonstrate that there are further metabolic processes in which a different source of carbon is used to facilitate tumor growth and proliferation. For instance, in pancreatic adenocarcinomas, glutamine is used as an energy source by CAFs to replace tricarboxylic acid cycle substrates [168]. Nonetheless, in ovarian and head and neck cancers, the study of different CAF populations among the same cancer types suggests a strong dependence on oxidative phosphorylation. This also supports the existence of metabolic heterogeneity [169,170]. Cells act on fibroblasts within the TME to indulge their needs for glutamine carbons, leading to an increased purine and pyrimidine biosynthesis, which further supports cancer growth. Analogously, the malignant cells supply lactate and glutamine to CAFs, resulting in the amplification of the TCA cycle and in enhancing the production of glutamine by CAFs [171]. Glycolysis and mitochondrial respiration in CAFs are promoted through a YAP/TAZ-dependent pathway, which represents a metabolic remodeling induced by ECM stiffening [164]. The high energy requirements of CRC cells are met by CAFs by overexpressing FASN and undergoing lipidomic reprograming, according to new research by Zhao et al. from 2020 [172]. A total of nineteen lipids that were generated and released by CAFs and ingested by CRC cells have been specifically identified [172]. The precise processes that trigger this lipidomic reprograming in CAFs and the manner in which cancer cells make use of the de novo lipids, however, require further investigation [173].

#### 2.4.5. Treatment Resistance

The application in CRC cells of the previously mentioned stimulation of cancer stemness through the paracrine interplay among CAFs and cells of numerous cancers revealed that CAFs can increase their stemness both in vitro and in vivo, according to mouse models, through the upregulation of netrin-1 as well as by transferring exosomal IncRNA H19 (acting as an inhibitor on stemness) [174].

Cancer stem cells play an important role both in tumor resistance and relapse. It has been revealed that increased secretion of cytokines by CAFs after chemotherapy resulted in enhanced resistance [175]. Specific chemotherapeutical regimes were found to be associated with higher resistance, such as oxaliplatin, 5-fluoracil and methotrexate, in CRC specimens where the increased presence of CAFs and CAF-derived exosomal miR-24-3p were demonstrated [176,177,178]. The existence of CAFs resulted in decreased sensitivity of malignant cells to cetuximab, which helps in the secretion of CAFs’ EGF, leading to resistance, as it is used in the treatment of metastatic CRC patients in combination with chemotherapy [179]. In addition to chemotherapy resistance, it has been found that CAFs-derived exosomes are responsible for resistance to radiotherapy, including exosomal miR-93-5p or miR-590-3p, which prevent malignant cells from apoptosis via radiotherapy. Additionally, these CAFs-derived exosomes also participate in advanced CRC stemness [180,181,182].

## 3. CRC-Associated Fibroblasts and Anti-Tumor Immune Response

### 3.1. CAFs and Tumor Immunity

CAFs, as major components of the stroma, remodel immunity, contributing to a chronic inflammatory state of cancer cells and modulating the immune responses to the tumor. This is fundamental for a tumor to survive [115,183]. The release of pro-inflammatory cytokines facilitates the macrophages, neutrophils and lymphocytes to be placed in tumor stroma. As they undergo differentiation into tumor-associated macrophages (TAMs) and tumor-associated neutrophils, within the TME, they release endothelial and growth factors (e.g., interleukins) [184,185]. In skin cancer, studies suggest that CAFs expressing the fibroblast activation protein (FAP)-positive marker diminish the cytotoxic function of T-lymphocytes [186] throughout the signaling pathways and tracks. This is followed by the lack of reaction to these factors, as mentioned above, eventually resulting in poor ability to regulate the immune responses. There is also a strong relationship between the presence of CAFs and tumor-infiltrating immune markers in various cancers, such as head and neck and lung cancer—for instance, the correlation between CAF and CD16-positive TAMs in α-SMA positive stained and CAFs marker FAP and CD14, respectively [43,187].

On the other hand, in mice models, by subtracting the FAP-positive CAFs and by inhibiting cytokines, such as SDF-1, the resulting immune control of tumor growth and the efficacy of immunotherapy are accelerated in pancreatic cancer [188]. In oral squamous cell carcinoma, cancer cells co-cultured with CAFs presented a higher expression of cancer-associated markers, which were used to identify neoplasms with macrophage lineages. Additionally, they were expressed by malignant cells from other lineages (e.g., antigens, such as CD68, CD163, CD14, CD200R, HLA-G, CD80 and CD86) supplementary to the expression of genes, such as ARG1, IL-10 and TFG-1β. These cells are revealed to suppress T-cell proliferation [189]. In esophageal cancers, Kato et al. [190] demonstrated with a series of in vivo and in vitro models that IL-6 mediates both the CAFs suppression of CD8^+^ and the promotion of FoxP3+ tumor-infiltrating lymphocytes, resulting in experimental blockage of IL-6, suggesting a promising efficacy of immunotherapy.

In CRC, the increased expression of specific CAF markers reflects their relationship with immune cells. CAFs play a significant role in tumor immunity; a-SMA expression has been found in greater quantities in CRC than in physiologic colonic mucosa, and their relation with tumor-infiltrating lymphocytes was found to be negatively correlated, whereas a different marker, called fibronectin, together with a-SMA was positively correlated in CRC [182]. An interrelationship with CD8 T cells and CAF phenotypes that substantiates the importance of CAFS was observed in CRC [191]. Confirming the positive correlation between PD-L1 in CRC, CAFs via the CXCL5 secretion participated in the PD-L1 expression [192].

Moreover, CAFs also promote the adhesion of monocytes by upregulating ICAM-1 and VCAM-1 expression in CRC cells [193]. A further immunosuppressive activity of CAFs is the secretion of IL-8, which brings monocytes to CRC tissues. Additionally, CAFs promoting macrophage M2 polarization lead to the suppression of natural killer (NK) cell activity [194].

### 3.2. Alternation of the Antitumor Immune Response by CAFs

CAFs, as a major component of the TME, modify the TME and affect both the innate and adaptive anti-tumor immune response as a result of their release of the mentioned cytokines, chemokines or other soluble substances [106,195,196,197]. The cytotoxic function and cytokine production of NK cells, as well as the susceptibility of tumor cells to NK-mediated lysis, are all affected by CAFs [198,199,200]. Additionally, CAFs promote the recruitment of innate immune cells, such as tumor-associated macrophages (TAM) [201] or potentially tumor-associated neutrophils (TAN) [202] and their acquisition of an immunosuppressive phenotype (M2 and N2, respectively) [201,203], and activate mast cells with a potential immunosuppressive phenotype [204]. CAFs interfere with the maturation and function of dendritic cells while favoring the recruitment and development of MDSCs and Tregs [205,206,207]. Additionally, CAFs may affect CD4+ T-helper (TH) lymphocytes, favoring tumor-promoting TH2 and TH17 responses, and inhibit CD8^+^ cytotoxic T-cell activation, function and survival [207,208,209,210]. CAFs appeared to extend the enlistment of monocytes into the CRC TME by means of different mechanisms. At first, the ICAM-1 expression and affinity for monocytes are elevated in CRC CAFs, which increases their interaction and prolongs monocyte residence in CRC tissues [193]. Moreover, through increasing VCAM-1 expression in CRC cells, CRC CAFs aid in the adherence of monocytes. Additionally, by secreting IL-8, CAFs can also draw monocytes [194]. Then, CAFs encourage macrophages to become M2 polarized to reduce the activity of natural killer (NK) cells in CRC, favoring the tumor immunity’s ability to defend itself [194].

The ability of CAFs to control the immune checkpoints in CRC is important. Notably, the CD73 expression in CAFs is increased via an A2B-mediated feedforward circuit triggered by tumor cell death, which enforces the CD73 immune checkpoint and subsequently blocks antitumor immunity in CAF-rich CRC. CAFs are the majority of cells expressing CD73 in human CRC tissues, a molecule acting as an immune checkpoint to suppress immune activation through the A2A receptor [211]. When considered collectively, these CAFs’ immunosuppressive effects on CRC have important clinical ramifications, making them prospective therapeutic biomarkers, as well as CRC targets.

## 4. CRC-Associated Fibroblasts—Therapeutic Implications and Clinical Outcomes

### 4.1. CAFs’ Prognostic Value in CRC

It is reasonable to assume that as long as CAFs play an important role in tumor growth, invasion, migration and metastasis, they will probably be a significant prognostic tool [8]. Different research groups have tried to prove this hypothesis in various cancers.

In CRC, CAFs promote invasion and metastasis through different pathways. Back in 2014, Berdiel-Acer et al. [212,213] demonstrated that the functional heterogeneity of CAFs, derived from the differences of CAFs with the normal colonic fibroblasts, may be of prognostic significance. The five genes derived from CAFs (CCL11, PDLIM3, AMIGO2, SLC7A2 and ULBP2) were associated with higher relapse risk in CRC [212,213]. Another pathway of great significance is the TGF-β signaling pathway [9]. High stroma-to-tumor ratio and high stromal gene expression in CRC have been linked to poor prognosis [214]. Moreover, high abundance of CAFs is associated with the tumor stage. It has been shown that the CAFs’ negative prognostic significance is mainly associated with stage III CRC [215].

CAFs in CRC secrete exosomes that can lead to proliferation and chemoresistance through different ways [4]. Exosomes seem to drive the proliferation and growth of cancer stem cells (CSCs), which are targetable by 5-fluorouracil (5-Fu) and oxaliplatin (OXA) [216]. The production of exosomes with elevated miR-92a-3p by CAFs is another way of chemoresistance, through enhancement of the epithelial-mesenchymal transition (EMT). This enhancement is achieved via the activation of the Wnt/β-catenin pathway [217]. Long non-coding RNA (lnRNA) H19 overexpression and exosomal transfer seem to be related to stromal stiffness and chemoresistance [218]. Additionally, CRC-associated lncRNAs contribute to resistance to OXA via CAFs expression and their exosomal transfer by activating the β-catenin pathway [219]. The exosomal transfer of miR-21 and Wnt ligands expressed by CAFs is related to invasion and chemoresistance [220,221].

Other mechanisms have also been correlated with cancer invasion, metastasis and poor prognosis in CRC. Expression of endoglin, Wnt2 and Wnt5a by CAFs has been correlated with poor prognosis through different mechanisms, such as promotion of angionegenesis [145,158,222,223]. Furthermore, the differentiation of mediated hepatic stellate cells into CAFs through the CXCR4/TGF-β1 pathway has been linked to liver metastasis [224]. Heichler et al. [122] suggested another way of promoting tumor growth through the STAT3 activation into CAFs by IL-6/IL-11. Zheng et al. [225] revealed the prognostic significance for CRC patients of a single-cell and bulk RNA sequencing, which identifies CAFs’ related signature. Generally, the expression of a-SMA, FSP1, FAP, ubiquitin carboxyl-terminal hydrolase L1 (UCH- L1), lysyl oxidase-like 2 (LOXL2), CD70 and c-type lectin domain family 3 member B (CLEC3B) is correlated to poor prognosis [156,226,227,228,229].

The co-expression of a-SMA, FSP1, FAP and CD163 in DCSIGN (M2 macrophage markers) seem to have a prognostic role, whereas CAFs-related genes, such as osteopontin (OPN), GREM1 and ISLR, showed a predictive value for FOLFIRI/bevacizumab therapy, negative and positive prognostic value, respectively [229,230,231,232]. Moreover, Ferrer-Mayorga et al. [233] reported a better outcome in CRC, and it is believed that this was mainly due to the protective effect of the active vitamin D metabolite 1,25(OH)2D3. At last, different prognosis has been associated with different CAFs’ expression, such as a-SMA, podoplanin and S100A4 [234]. Regarding rectal cancer, CAFs radiotherapy activated or detected after neoadjuvant radiotherapy is linked to poor prognosis and seems to be an independent negative prognostic factor [161,235,236].

### 4.2. Therapeutic Implications

All the pathways implicated in the role of CAFs and their prognostic significance could be a possible therapeutic target in various cancers. Their role could be summarized in six basic parts: tumorigenesis, proliferation, angiogenesis, immune response, stemness and resistance and metastasis [229]. Some of the pathways that are followed to achieve these six functions include: MyD88 signaling, STAT3 activation, PI3K-Akt, FGF-1/-3/FGFR4, HGF-MET, ERK5/PD-L1 and Wnt2 signaling [229].

CAFs-related tumorigenesis is the target of MyD88 signaling inhibitor, BMP signaling inhibitor, STAT 3 inhibitor, DNA aptamer targeting periostin, NF-κΒ signaling agonist, PKCζ agonist, function-blocking integrin antibodies and their conjugation [9,229,237]. M2 macrophage polarization is induced by MyD88 signaling in CAFs, leading to colitis-related CRC tumorigenesis [120]. On this basis, Xie et al. [238] attempted to use TJ-M2010-5, an inhibitor of this pathway, to prevent colitis-related CRC in mice. STAT3 inhibition in CAFs has also demonstrated an anti-tumor effect on CRC [239]. Regarding breast cancer, DNA aptamers targeting periostin seem to limit tumorigenesis [240].

Anti-CAF-related proliferation therapies include IL-6 antagonists, PI2K-Akt inhibitors, DNA aptamers targeting periostin, ERK5/PD-L1 inhibitors, FDF-1/-3/FGFR4 inhibitors and HGF-MET inhibitors [229].

Angiogenesis is targeted by VEGF antagonists, STAT3 inhibitors, IL-6 antagonists, Wnt2 neutralizing antibodies and FAP-targeted chimeric antigen receptor T cells [229,241]. Wnt2 neutralizing antibodies seem to recall the immune response to the tumor through the activation of dendritic cells [242] and FAP-targeted chimeric antigen receptor T cells, leading to depletion of FAP + CAFs, revoking the pro-angiogenic CAFs-related effect [241].

The reduced immune response to cancer through CAFs could be restored with PD-L1 inhibitors, CD70 neutralizing antibodies, CD73 neutralizing antibodies, Wnt2 neutralizing antibodies and/or T-cell-based immunotherapy [229,242,243]. Anti-CD70 antibodies reverse immunosuppression, which is caused by Tregs on tumor stroma. Tregs abundance is correlated with CD70 expressed by CAFs [244]. Regarding the role of FGFR in the interaction of tumor cells and TME, including CAFs, it is also reasonable to assume that FGFR inhibitors could indirectly target CAFs [245,246]. Data have shown that FGFR inhibition, for example FGFR1 inhibitors, can lead to immunomodulation by triggering T-cell-based antitumor activity [247].

The phenotype of stemness and subsequent chemoresistance can be reversed by targeting netrin-1, EGF, IL-17A and exosomal miR- 24-3p, lncRNA H19, miR- 93-5P, miR- 590- 3p, the axis CXCL12, CXCR4 and TGFβ, mainly by avoiding the EMT phenotype [8,229,248,249]. Finally, the inhibition of metastasis pathways related to CAFs might be a therapeutic option. Targeting PDGF receptor signaling, FGF1/FGFR3, FAK pathway, HGF secretion, endoglin, activin A, Wnt2 and fatty acid oxidation, matrix metalloprotease 9 (MMP9) and hedgehog (Hh) signaling could delay tumor growth.

However, no treatment has succeeded in showing a clear statistical or clinical benefit for any cancer type until now, and further research is necessary [8,229].

## 5. In Vitro and In Vivo Models to Study CAFs

The current models include in vivo and two- and three-dimensional in vitro models (Matrigel, hydrogels, co-cultures with tumor organoids) (Table 2). In vivo, a set of all RNA transcripts of CAFs is not enough to restate the CAFs heterogeneity in the two-dimensional cultures [250,251,252]. Although three-dimensional matrices are significantly useful in the enrichment of our knowledge about the transcriptomes and phenotypes of CAFs [252,253,254,255], there are several limitations. By using such matrices, the identification of CAFs may not be entirely recapitulated. This leads to a limited investigation of the crosstalk between different cell populations in vitro. Models that use the ECM produced by CAFs [256] or maintain all cell populations within the tumor (such as the liquid–air interface) and microfluidic culture (such as organs-to-chips) could be used in a study of CAFs in vivo [257,258,259,260,261].

In terms of the in vivo models, although GEMMs for lineage tracing help us demonstrate the origin, heterogeneity, plasticity and roles of fibroblasts in wound healing and normal and inflammatory tissues, they fail in CAFs demonstration [262,263,264,265,266,267]. In addition, the dissection of CAFs by recombinase-based models could be useful in the study of CAFs in vitro [268]. The application of microscopy imaging techniques in GEMMs for CAFs could also play a part in understanding the origin and dynamics of CAFs [254,269].

Using publicly available information, a recent study examined the quantity and phenotype of cells marked by ASMA, PDGFR and FSP1 to investigate the relationship between CAF marker expression in tumors from MMTV-PyMT mice and humans in breast cancer. In human tumors, the expression of each CAF marker was heterogeneous and varied from patient to patient. However, there was a striking resemblance between the tumors from MMTV-PyMT mice and the consensus staining pattern for ASMA, PDGFR and FSP1. Cells expressing ASMA and PDGFR were found in fibrotic streaks with a spindle-shaped morphology, whereas FSP1+ cells had a rounded shape and were found in both the overt stroma and tumor cell clusters. Overall, none of the markers utilized in this study identified all CAF subsets. However, it was discovered that the distribution and morphology of stromal fibroblasts expressing ASMA, PDGFR and FSP1 in mouse and human breast tumors were strikingly similar [270].

**Table 2:** Culture models for studying CAFs.

Culture Model	References
Generation of cancer spheroid and 3D mucosal sheet model	[271,272,273,274,275,276]
Cell viability assays	
Visualization of hypoxia in the 3D cell-sheet model	
Green fluorescent protein gene transfection	
Reverse transcription-quantitative polymerase chain reaction and immunoblotting	
**CAFs sources**	
Primary CAFs (mouse/human)	[277,278]
LX-2	[279,280,281,282,283,284,285,286]
Primary HSCs	[287,288]
3T3-NIH	[288]
**Cell lines**	
HCT-116	[224,279]
LS174T	[279]
HT-29	[224,289]
CT-26	[287,288]

## 6. Conclusions

Although a great therapeutic progression has been achieved in the cancer field, and the screening methods are more widespread, it remains one of the major health issues. Tumor stroma has been examined for tumor growth, invasion, metastasis and resistance to therapy. An important component of tumor stroma are CAFs. CAFs, along with the rest of the TME, interact with cancer cells, leading mainly to tumor progression. This interaction occurs through many ways, such as exosomal transfer. EMT transformation is one of the main ways by which normal cells turn to different CAFs types. Different CAFs have different functions, promoting tumor progression through various pathways.

CRC, in comparison to other malignancies, has a better prognosis and treatment response. However, in recent years, little progress has been made in therapeutic options, especially in the metastatic setting. In addition, only <15% of CRC is characterized as metastatically high and can earn the benefit from the immune checkpoint inhibitors. Regarding the molecular classification of CRC, CMS4 has been correlated with a worse prognosis. CMS4 is linked to mesencymal transformation and high stromal gene expression.

According to the above and taking into consideration the role of CAFs in CRC development, growth, invasion, metastasis and treatment resistance, their detection, characterization and functional understanding is of great significance for reversing tumor growth and chemoresistance, leading to greater treatment options. Different markers have been used for the detection and characterization of CAFs in the CRC setting, and different targeted therapeutic molecules are under evaluation. However, there is no consensus for their use, and no benefit has been proven.

More preclinical and clinical research remains to be performed to clarify the CAFs’ mechanisms of action and their role as a potential therapeutic target in CRC.

## Figures and Tables

**Table 1:** Markers expressed in CAFs of different cancers.

Cancer	Markers	References
Lung	α-SMA, osteopontin	[63,64,65,66]
	VCAM-1	
	Stemness factors: nanog/Oct4	
Breast	α-SMA, FAP, PDGFR-α/β, CD29, NG2, FSP1, vimentin, PDPN	[67,68,69,70,71]
Gastrointestinal	FAP, α-SMA, vimentin, FSP1, PDGFR-α/β, periostin	[72,73,74,75,76,77,78,79]
Skin	α-SMA, FAP, vimentin, PDGFRα	[80,81,82]
Ovarian/Endometrial	α-SMA, FAP, FSP1, FGF-1, vimentin	[83,84,85]
Head and Neck	α-SMA, PDPN, FAP, PDGFR-α, PDGFR-β, FSP1, NG2	
Genitourinary	α-SMA, vimentin, FAP, FSP1, PDGFR-α, PDGFRβ, CD90, MFAP5, POSTN	[86,87,88,89]
CRC	α-SMA, FAP, FSP-1, PDFRα, PDFRβ, CD10, IL-11, ADAMs, exosomal mcRNAs, Tenascin C, ED-A FN, SDF1, LTBP2, CDH11, OLFML3, FSTL1	[86,87,88,89]

## Data Availability

Not applicable.
